# Effects of Guanidinoacetic Acid and Metabolizable Energy Levels on Performance and Nutrient Metabolism in Broilers

**DOI:** 10.3390/ani16060935

**Published:** 2026-03-16

**Authors:** Patrícia Tomazini Medeiros, Edenilse Gopinger, Everton Luis Krabbe, Victor Naranjo, José Henrique Stringhini, Alex Maiorka

**Affiliations:** 1 Veterinary Sciences, Federal University of Paraná, Curitiba 80035-050, Brazil; amaiorka@ufpr.br; 2 Animal Science, Stimulus Fellow the Innovation of the Edmundo Gastal Agricultural Research and Development Support Foundation, Concórdia 89715-899, Brazil; edezoo@yahoo.com.br; 3 Brazilian Agricultural Research Corporation-Embrapa Swine and Poultry, Concórdia 89715-899, Brazil; everton.krabbe@embrapa.br; 4 Evonik Guatemala, Guatemala City 01010, Guatemala; victor.naranjo@evonik.com; 5 Department Animal Science, Federal University of Goiás, Goiânia 74690-900, Brazil; henrique@ufg.br

**Keywords:** metabolizable energy, guanidinoacetic acid, broiler performance, nutrient digestibility

## Abstract

The effects of three metabolizable energy (ME) levels and the use of guanidinoacetic acid (GAA) were evaluated on broiler performance and nutrient digestibility from 1 to 35 d of age. In total, 1944-d-old Ross AP95 male broilers were randomly distributed to six treatments (12 replicates/treatment). Diets were formulated to contain three ME levels (standard energy [SE], −50 kcal/kg reduced energy [−50 RE] and −100 kcal/kg reduced energy [−100 RE]) in all feeding phases with or without GAA inclusion. For the nutrient-metabolizable analysis, 960-day-old male broilers were separately raised in floor pens until 14 d of age and randomly distributed to six treatments (16 replicates/treatment). Data were analyzed with ANOVA and Tukey’s test at *p* ≤ 0.05. There was a significant interaction for the feed conversion ratio (FCR) at 21 days, in which the PC diet showed better FCR when GAA was included. In the evaluation of the main effects, an effect of metabolizable energy (ME) was observed on body condition score (BCS) at 7 and 21 days, feed intake (FI) at 21 and 35 days, and feed conversion ratio (FCR) at 21 days, with the PC diet showing better FCR and lower FI. An effect of GAA was observed on feed conversion ratio at 21 days, with the inclusion of GAA in the diet showing better FCR. In conclusion, broilers fed SE diets with GAA, beyond better performance, had improved AME and AMEn compared to broilers fed RE diets without GAA in starter diets.

## Simple Summary

This study evaluated how different metabolizable energy levels in diets and the addition of guanidinoacetic acid (GAA) affect the performance and nutrient utilization of broiler chickens. Diets with standard energy, reduced by 50 kcal/kg, and reduced by 100 kcal/kg were tested with or without GAA. The results showed that GAA improved feed conversion and energy utilization in diets with standard energy, especially in the starter phase (1 to 21 days). However, GAA did not fully compensate for the energy reduction in diets with reduced 50 kcal/kg or 100 kcal/kg calories. Digestibility analyses showed greater apparent metabolizable energy (AME and AMEn) in broilers that received GAA in normal diets, but there were no major effects on protein or mineral digestion. The inclusion of GAA may improve the performance and energy efficiency of broilers in the starter phase, mainly when used in diets with adequate energy, helping to improve nutrient use.

## 1. Introduction

Guanidinoacetic acid (GAA) is synthesized endogenously from L-arginine and glycine, primarily in the kidney and pancreas. One gram of GAA can replace 1.49 g of arginine in broiler diets [1]. Once GAA is formed, it is transported to the liver, where it is methylated to form creatine. Creatine is then distributed via the bloodstream to tissue with high and fluctuating energy demands, such as skeletal muscle. Creatine functions in cellular energy metabolism and interacts with the adenosine triphosphate (ATP) system in muscle, which is the primary energy source used for maintenance and growth [1,2,3,4].

Therefore, GAA supplementation increases muscle creatine and phosphocreatine stores by up to 21% with supplementation rates registered for use, thereby enhancing the capacity for rapid ATP regeneration via the creatine kinase reaction [5]. The phosphocreatine–ATP system acts as an energy buffer, supporting cellular energy homeostasis during periods of high demand, such as muscle contraction and growth [2,4,5]. The improved energy transfer efficiency leads to better feed conversion and energy utilization, particularly when dietary metabolizable energy (ME) is adequate [4,5,6]. When dietary ME is reduced, GAA can compensate the energy deficiency by optimizing cellular energy metabolism; however, it may not fully offset substantial energy deficits under optimal management conditions [4,5,6]. Thus, the physiological basis for the interaction between GAA and dietary ME lies in the enhanced ability of broilers to maintain ATP levels and support growth, especially under conditions of limited energy supply [5,6].

However, creatine itself is not practical to use as a feed additive due to its relative instability and has not been authorized as a feed additive [1,7,8]. Furthermore, broilers are typically fed vegetable-based diets, and the supply of exogenous creatine is reduced to virtually zero [4,9]. GAA is considered an effective alternative source of creatine in broiler diets [1,4,5]. Creatine is chemically unstable under typical feed-manufacturing conditions: thermal and moisture exposure promotes non-enzymatic cyclization of creatinine. Quantitatively, low recoveries have been documented under retorting/extrusion and during storage (e.g., 36% after retorting, 85% after extrusion, and a 63% decline during 15 months at 25 °C), illustrating the difficulty of guaranteeing creatine content in finished feeds [10]. Reviews consistently report poor creatine stability during pelleting, whereas GAA is thermally stable and suited to pelleted diets [1,5,7].

Moreover, under the EU feed-additive framework (Regulation (EC) 1831/2003), only authorized additives may be used. The EFSA has issued a positive opinion on guanidinoacetic acid (GAA) as a performance-improving additive in chickens for fattening (≥600 mg/kg), while creatine itself has no corresponding EU feed-additive authorization in the European Commission’s feed additives register [11].

Due to its biological functions, GAA has better potential for inclusion in practical broiler diets, as it is more stable and cost-efficient than creatine [6]. It has been shown to improve broiler performance, particularly during the growth phase [5,6]. Improvements in feed conversion ratio (FCR) have been observed mainly in the finisher phase with the inclusion of 0.6 and 1.2 g/kg of GAA under heat-stress conditions [12]. Khajali et al. (2020) [5] reported that supplementation with 0.6 g/kg of GAA improved FCR by 2.55% [4].

Several studies [4,6,13] have shown that supplementation with GAA in broiler diets can compensate for a reduction of 50 kcal/kg and 88.5 kcal/kg in metabolizable energy (ME). Furthermore, the energy contribution of GAA in the nutritional matrix of diets can lead to different economic benefits depending on raw material costs, which vary across countries and regions. This improvement in energy utilization enables a sparing effect on dietary energy sources, effectively reducing the overall energy demand of the diet. The inclusion of GAA in poultry diets can lead to a reduction in the dietary energy content without compromising growth performance, thereby offering a cost-saving potential in feed formulation [14]. GAA supplementation may reduce feed costs by optimizing the energy efficiency of the diet [15]. These findings suggest that GAA can serve as a strategic component in the nutritional matrix of animal feeds, providing an economically advantageous approach by potentially decreasing the inclusion of costly energy-dense ingredients. This in turn contributes to lower feed costs and improved economic sustainability in animal production systems.

Information regarding the effects of reduced metabolizable energy (ME) levels combined with GAA supplementation on apparent metabolizable energy (AME) are contradictory regarding ME compensation. Therefore, the aim of this study was to evaluate the effects of GAA inclusion in diets with different levels of metabolizable energy on poultry performance, apparent metabolizable energy, and nutrient digestibility.

## 2. Materials and Methods

### 2.1. Animal Care

This broiler trial was conducted at the Experimental Poultry Sector of Embrapa Pigs and Poultry—CNPSA, located in Concórdia, Santa Catarina, Brazil. All procedures involving live birds were reviewed and approved by Embrapa’s Animal Ethics Committee under protocol 017/2022.

### 2.2. Bird Husbandry

A total of 1944-d-old male Ross AP95 broilers with an initial average weight of 41.9 g were obtained from a commercial hatchery. Birds were housed in a dark house system facility and allotted 1.70 × 1.60 m floor pens, each equipped with tubular feeders, five nipple drinkers, and new pine wood shavings. A total of 72 pens were used, with 12 per treatment and 27 birds per pen. The research facility featured a controlled environment with a negative pressure ventilation system, exhaust fans, inlets vents, evaporative cooling pads, forced-air heaters, and a control panel for temperature and ventilation management. Up to 25 days of age, the birds received 24 h of light with an intensity of 40 lux and heating provided by brooders with lamps. After this period, the birds received 16 h of light with an intensity of 20 lux and 8 h of darkness. Feed and water were provided ad libitum. The house temperature was set at 33 °C at placement and gradually decreased to 18.3 °C by day 35 to ensure bird comfort and optimal performance.

### 2.3. Feed Formulation and Experimental Design

All birds were weighed and distributed to pens following a completely randomized design. A 3 × 2 factorial arrangement was used to evaluate three dietary metabolizable energy levels and two levels of GAA inclusion, with 12 replicates per treatment. The ME levels included a standard energy (SE) diet, formulated according to Aviagen recommendations for Ross AP95 broilers (starter = 2980 kcal/kg, grower = 3050 kcal/kg, finisher = 3100 kcal/kg), and two reduced energy (RE) diets with reductions of 50 kcal/kg (−50 RE) and 100 kcal/kg (−100 RE) relative to the SE diet [16]. The GAA factor consisted of either the inclusion of 600 g/ton of GAA or no inclusion. The following nutritional matrix was considered for GAA: 221% crude protein, 77% arginine and 83,000 kcal/kg of sparing AMEn (apparent metabolizable energy corrected for nitrogen balance), corresponding to an energy contribution of 50 kcal to the diet. The GAA product (GuanAMINO^®^, Evonik Operations GmbH, Hanau-Wolfgang, Germany) utilized in this study had a purity of ≥96% specified by the manufacturer. Furthermore, the GAA product was provided in a granular form achieved through granulation with starch (approx. 1% inclusion) to ensure dosing accuracy and homogeneous distribution in the feed.

The experimental diets were formulated to meet all the nutritional requirements recommended by Aviagen (2022) [16] and are presented in Table 1. Broilers were fed using a 3-phase feeding program: starter (1–7 d), growing (8–21 d) and finisher (29–35 d). Diets were pelleted and crushed until day 12, and as pellets from day 13 until the end of the experiment.

### 2.4. Growth Performance

At 1, 7, 21, and 35 days of age, bird body weight (BW), feed intake (FI), and mortality were recorded to calculate body weight gain (BWG), feed conversion (FCR; average feed intake/average weight gain), and caloric conversion (CC; metabolizable energy intake/body weight gain). Mortality was also recorded daily to adjust average feed intake and total body weight gain. Mortality was recorded by weight and date of death. Weight gain was adjusted by the formula: average weight gain (live) + weight at death.

Feed consumption was adjusted by the formula: total consumption/number of live birds + bird factor. The bird factor was calculated by dividing the number of days the birds consumed feed by the number of days in the period.

### 2.5. Nutrient Digestibility

For the nutrient digestibility trial, birds from the same hatchery as those used for the performance trial were raised separately in floor pens with pine-shaving bedding until 14 days of age. Subsequently, they were transferred to battery cages equipped with grated floors and collection trays. The birds were distributed in a completely randomized design using a 3 × 2 factorial arrangement (3 metabolizable energy levels with and without GAA) with 16 replicates per treatment, totaling 96 experimental units, with 10 birds each.

Birds were submitted to a 4 d adaptation period to the facilities and experimental diets, followed by 5 days of total excreta collection. Nutrient digestibility was evaluated in birds from 18 to 22 d of age [17]. During this period, excreta were collected daily, packaged, and frozen (−20 °C) to prevent sample fermentation prior to subsequent processing.

At the end of the collection period, samples were thawed at room temperature, weighed, and homogenized with feathers and other contaminants removed from the samples. A 500 g aliquot was separated and oven-dried with forced ventilation at 60 °C for 72 h for further analysis and calculation of nutrient digestibility of dry matter (DM), crude protein (CP), and calcium and phosphorus balance. Nitrogen balance was determined by the difference between nitrogen intake and nitrogen excreted. Gross energy was determined in a calorimetric bomb (Parr6400 Automatic Isoperibol Calorimeter, Moline, IL, USA). Dry matter (DM) content was determined by oven-drying at 105◦ C for 12 h, and the crude protein was analyzed according to method 954.01 [18].

During this period (18 to 22 d of age), diet samples were also collected to determine the apparent nutrient digestibility coefficient (ADC). The following formula was used: ADC (%) = ((NC − NEx)/NC) × 100; with ADC = apparent nutrient digestibility coefficient (%); NC the amount of the nutrient consumed; and NEx the amount of the nutrient excreted. Based on this, the apparent nutrient digestibility coefficients of DM and CP were calculated. From the gross energy values, apparent metabolizable energy (AME) and apparent metabolizable energy corrected for nitrogen (AMEn) were also determined [19].

To determine the amount of nutrients consumed and excreted, feed consumption was fully monitored by recording both the total amount of feed offered and feed refusals. The excreta were also measured, with the total amount produced during the collection period being collected and weighed [17].

To calculate the amount of nutrient consumed, the following formula was used: NC = TFC × NFA; where NC is the amount of nutrient consumed; TFC is the total feed consumed during the period; and NFA is the amount of nutrient in the feed. Similarly, the amount excreted was calculated using the formula Nex = TPEx × NExA, where Nex is the amount of nutrient excreted, TPEx is the total excreta produced, and NexA is the amount of nutrient in the excreta.

### 2.6. Statistical Analyses

Data were analyzed using a 3 × 2 factorial arrangement (ME level × GAA inclusion) in a completely randomized design to evaluate main effects and interactions and evaluated by an age-independent model, considering each pen as an experimental unit. Initially, data were tested for normality using the Shapiro–Wilk test, followed by ANOVA [20] to assess the interaction between ME levels and GAA inclusion. When a significant interaction was detected, it was further explored by comparing the effect of GAA inclusion within each ME level and vice versa. When there was no significant interaction, main effects were evaluated separately: ME levels were compared using Tukey’s test, and GAA inclusion was assessed using a *t*-test, both at a 5% significance level.

Performance data (live weight, feed intake, and feed conversion ratio) were analyzed separately for each evaluation period (7, 21, and 35 d) using ANOVA in a 3 × 2 factorial arrangement. Each analysis considered the main effects of ME level and GAA inclusion and their interaction. When a significant interaction was detected, the factors were broken down. The effects were evaluated separately: the ANOVA F-test was used to determine significant effects of GAA (*p* ≤ 0.05) and Tukey’s test to determine significant effects of ME level.

## 3. Results

### 3.1. Broiler Performance

Overall, the average growth performance observed in this experiment notably exceeded the Ross AP95 performance targets. A significant interaction was observed between the factors for feed conversion at 21 days. For the other performance variables, no interaction (*p* > 0.05) was found between the reduction in metabolizable energy (ME) content and GAA inclusion on bird performance from days 1 to 7, 1 to 21, and 1 to 35 (Table 2).

Evaluating the interaction between ME level and GAA inclusion, a significant effect on feed conversion was observed, where both with and without GAA a reduction in energy in the diets impaired conversion, with the positive control showing the best response. When evaluating the effect of GAA use within each energy level, an effect was observed only in the PC diet, where GAA inclusion improved feed conversion (Table 3).

Regarding the main effects, body weight (BW) at 7, 21, and 35 days was not significantly affected by either ME level or GAA inclusion. Body weight gain (BWG) was not affected by ME levels, regardless of GAA inclusion, at either of the evaluated ages.

In the evaluation of the main effect, an ME-level effect was observed on caloric con-version at 7 days, feed intake at 21 and 35 days, and CC at 21 days (Table 4). The feed intake, an effect of ME level, was observed in diets, where the positive control diet resulted in lower intake compared to the RE-100 diets in 21 and 35 d.

At 7 and 21 days, the positive control had a greater CC, differing from the diet with −100 kcal/kg (Table 4). When evaluating the effect of GAA inclusion, no significant differences in body weight were observed at 7, 21, or 35 days. Feed intake and weight gain were not influenced (*p* > 0.05) by GAA inclusion within any ME level and age.

Regarding the overall effect of GAA inclusion, birds supplemented with GAA presented better CC (*p* = 0.0004) at 21 days of age compared to the non-supplemented birds (Table 5).

### 3.2. Nutrient Digestibility

The *p* values for main effect ME and GAA and interaction ME X GAA from the metabolizable assay are presented in Table 6. No significant interaction or main effects were observed for the crude protein (CP) digestibility coefficient or nitrogen balance. However, a significant interaction (*p* <0.05) between metabolizable energy (ME) levels and GAA inclusion was detected for apparent metabolizable energy (AME), nitrogen-corrected apparent metabolizable energy (AMEn), calcium digestibility coefficient (DC Ca), phosphorus digestibility coefficient (DC P), and dry matter digestibility coefficient (DC DM) (Table 7 and Table 8).

When evaluating AME and AMEn (Table 7), it was observed that both GAA-supplemented and non-supplemented diets led to a reduction in AME and AMEn values. However, when evaluating the effect of GAA inclusion within each dietary energy level, a significant effect was observed only in the positive control diet, where GAA supplementation resulted in higher AME and AMEn values compared to the non-supplemented counterpart.

Within diets without GAA, phosphorus utilization was higher in the −50 RE diet, which differed significantly from the positive control diet. When evaluating the effect of GAA inclusion at each energy level, GAA supplementation in the −50 RE diet resulted in reduced phosphorus utilization (Table 8).

Regarding the dry matter (DM) digestibility coefficient (Table 9), the −100 kcal/kg ME diet with GAA supplementation showed the lowest digestibility among the energy levels with GAA. For the effect of using GAA within each energy level, the −50 kcal diet with GAA presented a higher DM digestibility coefficient compared to the diet without GAA supplementation. The −100 kcal diet with GAA showed a lower DM digestibility coefficient than the diet without GAA supplementation.

## 4. Discussion

Performance results demonstrated the effect of GAA on feed conversion when supplemented in diets formulated with recommended energy levels (PC diet) during the starter phase (1–21 days) of broilers. The starter phase has been identified as a critical period for body growth, with birds showing high responsiveness to nutritional enhancements in the early days of life [7,21]. An important aspect to consider is the limited capacity of young birds to utilize lipids efficiently during this phase [22,23]. Notably, the positive control supplemented with GAA during the starter phase (1–21 days) had an improved FCR.

Consistent with these principles, in the present study (thermoneutral environment), broilers increased feed intake on reduced-energy diets (Table 2), yet AME/AMEn still decreased with lower energy across treatments (Table 7), indicating that energy per kilogram of feed remained limiting even as birds compensated partly via intake. Under thermoneutral conditions and good management, broilers typically adjust feed intake in response to dietary energy density such that moderate ME reductions can be partly compensated by higher intake when balanced protein (SID amino-acid ratios) and feed form are maintained. This intake-driven regulation and the need to preserve daily amino-acid supply are emphasized in broiler nutrition guidance and energy-management reviews [16,24,25].

In the starter cohort (1–21 d), FCR was improved by 0.04 when 0.06% GAA was included in the diet [8]. DeGroot et al. (2019) observed a 6% increase in BW and BWG in birds fed diets supplemented with 0.12% GAA at 14 and 28 days of age [2].

The improvement in FCR observed with the diet with higher metabolizable energy levels can be attributed to oil supplementation, which provides an extra caloric effect. This effect enhances nutrient availability and contributes to improved energy efficiency by increasing the net energy content of the feed [22].

Performance results for the overall period (1 to 35 days) demonstrated the effect of energy level on feed intake, with reduced consumption observed in diets with higher ME. Voluntary feed intake in broilers is regulated by energy intake, so increasing dietary energy levels can result in a reduction in feed intake [26].

Therefore, a reduction in the energy value of the diet results in an increase in feed intake, a fact also observed in this study. However, the increase in feed intake in low-energy diets is constrained by the birds’ physical capacity, which may override energy regulation mechanisms [27]. In this study, it can be inferred that no physical limitation affected feed intake, because there was no significant difference in weight gain.

Supplementation with 0.6 and 1.2 g of GAA/kg in diets formulated either with recommended energy levels or reduced energy levels improved the feed conversion ratio (FCR) of broilers in both cases, with a more pronounced effect observed in the low-energy diets [6]. The authors attributed the positive effects of GAA on performance to its essential role as a precursor of creatine and its ability to increase muscle creatine and ATP reserves. Creatine plays the role of a transporter in the form of phosphocreatine, enhancing energy supply in tissue with high energy demands, such as muscle and heart [6].

The data from the present study show the effect of GAA when used on top of energy-reduced diets did not produce significant effect. Previous studies demonstrated that GAA supplementation can improve cellular energy status [10]. Dietary GAA contributed to modulate creatine metabolism by increasing serum creatine kinase activity and arginine concentration to improve the efficiency of energy utilization [28].

Reports on AME responses to GAA are mixed and appear context-dependent: in some studies: GAA did not alter AME under the conditions tested [6,13,15], whereas in the present study AME was significantly higher in the PC + GAA than in the PC group (Table 4). This apparent discrepancy likely reflects differences in dietary energy density and lipid environment, age/phase and assay methodology (e.g., AME vs. AMEn; total-tract vs. alternative approaches), and matrix interactions with minerals and enzymes. In our dataset, the AME increase was restricted to the positive-energy control and was not observed in the −50 or −100 kcal/kg diets—consistent with a conditional energy benefit that depends on diet context [6,13,15,25,29].

The beneficial effects of GAA are most prominent under low-energy conditions and animal product-free diets, where energy availability may limit growth in broilers with high metabolic demands [6,30]. However, in the present study, GAA supplementation did not compensate for the reduction in dietary energy, possibly due to the optimal management and environmental conditions under which the birds were raised [25,29].

The reduction in dietary energy by 50 and 100 kcal/kg combined with 0.06% GAA supplementation showed no significant effects on AMEn or dry matter digestibility coefficients (DM DCs) in broilers [4,31]. Similarly, GAA supplementation did not affect AME values in broiler diets, as previously reported [6].

In a study where dietary energy was reduced by 50 kcal/kg and supplemented with 0.06% GAA, higher net energy values were observed compared to the same energy-reduced diet without GAA supplementation [28]. Additionally, GAA supplementation has been shown to increase net energy for production (NEP), which may lead to greater energy retention as fat in broilers [6].

Although dietary metabolizable energy is widely used to express the energy concentration in poultry diets, diets with the same ME are not necessarily utilized with equal efficiency by birds [4]. The improvement in feed conversion ratio (FCR) resulting from GAA supplementation may be attributed to enhanced cellular energy metabolism, as a 0.6 g/kg inclusion of GAA has been shown to increase muscle creatine by 15%, positively impacting energy utilization [5,25]. In addition to performance improvements, GAA supplementation has been associated with significant economic benefits. The inclusion of GAA in broilers could be an effective strategy to improve growth performance and gut function, which are critical parameters for economic efficiency in poultry production [32]. Beyond its energy-sparing effects, GAA also contributes to cost-effectiveness by potentially reducing the dietary requirement for arginine, a relatively expensive amino acid, thereby lowering overall feed costs [33]. Thus, the integration of GAA into poultry feed formulations offers a dual advantage: improving performance metrics and delivering economic gains through its nutritional contribution.

Although the diets were formulated with a GAA matrix value of ~50 kcal/kg AMEn (0.06% GAA), matrix values are engineering assumptions based on average energy equivalence reported in prior studies and do not guarantee a measured AME/AMEn gain under all contexts [4,9]. In our trial, GAA increased AME/AMEn only in the positive-energy control (PC) (Table 4), but failed to reach significance in the −50 and −100 kcal/kg diets for several context-dependent reasons: (i) lipid environment—lowering soybean oil to achieve ME reduction compromises emulsification and passage rate, conditions under which enterocyte benefits from creatine-supported ATP buffering are blunted, while energy responses to diet energy are well known to depend on lipid level/quality and feed form [23,34]; (ii) ME vs. NE handling—the matrix is expressed on an AMEn basis, yet the net energy contribution of added fat is disproportionately higher than starch because lipids have a lower heat increment, and when oil is replaced by cereals, birds may increase intake, but the realized NE can still be limiting, attenuating the apparent “50 kcal” sparing in vivo [16,24]; (iii) methyl-donor economy—conversion of GAA to creatine requires SAM-dependent methylation, and if methyl-donor supply (methionine/folate/betaine) is marginal, creatine loading and downstream ATP buffering may be reduced, limiting the energy effect in leaner (lower-oil) diets [35,36]; (iv) mineral–phytase interactions—Ca:aP and phytase level modulate nutrient utilization: fixed Ca:aP and a single phytase dose can alter AME/AMEn responses across energy strata and may partially offset a small matrix value [33,37]; and (v) intake compensation and management factors—under thermoneutral conditions, broilers compensate for lower ME by increasing feed intake, which helps maintain growth, but still results in lower AME/AMEn per kilogram of feed. When feed intake is constrained (e.g., by pellet quality, bulk density, or temperature), integrators typically increase nutrient density rather than rely on intake compensation [16,31,38,39].

These factors may explain why the formulation value (50 kcal/kg AMEn) did not completely translate into a significant measured energy-sparing effect in the reduced-energy diets, while the PC + GAA treatment—where emulsification and energy density were optimal—did show higher AME/AMEn and improved early-phase energy efficiency (Table 2 and Table 4), consistent with studies demonstrating GAA benefits under adequate dietary energy and non-stress conditions [2,13,28].

Additionally, guanidinoacetic acid (GAA) is methylated to creatine by guanidinoacetate N-methyltransferase using S-adenosyl-methionine, and creatine fuels the phosphocreatine shuttle that buffers ATP at sites of high energy demand [25,29,40]. In epithelia, including intestinal mucosa, the creatine transporter (SLC6A8) is expressed and regulates epithelial energy balance and barrier function. Altered transporter activity affects ATP availability and epithelial integrity [25,30,41,42]. In this context, a plausible linkage from the energy and mineral findings to cell physiology is that improved ATP buffering in enterocytes supports the activity of ATP-dependent pumps and Na^+^-gradient-driven cotransporters during nutrient absorption. For phosphate (Pi) absorption, the apical Na^+^-dependent transporter NaPi-IIb (Slc34a2) in the duodenum is the principal active pathway in chickens. Its function relies on the transmembrane Na^+^ gradient maintained by basolateral Na^+^/K^+^-ATPase [42,43]. Sustaining a high local ATP–ADP ratio (via creatine/phosphocreatine) stabilizes Na^+^/K^+^-ATPase activity and thereby favors NaPi-IIb-mediated Pi uptake and basolateral efflux [44,45]. For transcellular Ca^2+^ movement, apical entry (e.g., TRPV6), cytosolic buffering (calbindin-D28K), and basolateral extrusion (PMCA1b/NCX) ultimately depend on ATP supply and/or the Na^+^ gradient; thus, improved enterocyte energy status can enhance Ca utilization as well [44,46].

These mechanisms align with the digestibility results: in the positive-energy control (PC) diets, GAA increased AME/AMEn and P utilization (Table 4 and Table 5). Under PC conditions, higher lipid content improves emulsification, micelle formation, and passage rate, amplifying the payoff from ATP-buffered transport at the brush border [23,34]. By contrast, in the −50 kcal diet, P utilization fell with GAA. Two non-exclusive explanations are that reducing oil can blunt emulsification and shorten retention time, limiting the functional gain from enhanced ATP buffering [23,34], and GAA methylation draws on the methionine–folate–betaine cycle: if methyl equivalents are marginal, transient SAM/SAH and homocysteine shifts can modulate transporter expression or epithelial redox regulation [3,35,47]. These ideas are testable and motivate the targeted measurements proposed below.

Finally, an improved mucosal morphology may contribute, as several studies reported villus height and VH:CD improvements with GAA or with higher dietary energy, increasing absorptive area and potentially mineral capture, although responses are not universal [25,29,32,36]. This variability reflects the heterogeneous Ca utilization across the different energy levels.

Overall, the data support an energy-dependent mechanism: with adequate dietary energy and lipid emulsification (PC). GAA-driven creatine loading enhances enterocyte ATP buffering, supporting Na^+^/K^+^-ATPase-dependent Pi transport and ATP-dependent Ca^2+^ extrusion and thereby increasing AME/AMEn and P utilization. When energy is constrained, the lipid environment and methyl-donor economy may limit these benefits or yield neutral/negative effects on specific mineral coefficients [4,5,23,40,47].

In commercial corn–soy–soybean oil diets that do not contain inert fillers, crediting a 50 kcal/kg AMEn reduction via a GAA matrix (0.06% inclusion; matrix ≈ 83,00 kcal/kg AMEn for GAA) typically allows a ~0.4 percentage-point (pp) decrease in soybean oil and a ~0.4 pp increase in corn, with small downward adjustments to soybean meal and crystalline amino acids to preserve SID amino-acid ratios to account for the slight protein increase from replacing oil with corn [16]. A fixed replacement rate should not be applied across formulations because ingredient energy values vary by origin, age of birds, moisture, and analytical method. Instead, the adjustment should be solved with current ingredient matrices in a lowest-cost optimization calculator [16]. The feasibility of the 50 kcal/kg reduction is driven by the large AMEn gap between soybean oil (≈8700–8800 kcal/kg) and corn (≈3300–3350 kcal/kg) [45,46]. In most price decks, this substitution pattern reduces total feed cost by ≈1–2% after accounting for the cost of GAA inclusion, because soybean oil carries a substantially higher cost per unit of available energy than cereals: when a 100 kcal/kg reduction is implemented (≈ −0.8 pp oil/+0.8 pp corn, rebalanced AAs and soybean meal), up to ≈4% feed-cost reduction is commonly observed, contingent on local ingredient prices and constraints [16,31,48,49].

### Study Limitations and Future Research Directions

Several physiological and environmental factors my elucidate why certain parameters, such as growth performance did not improve in some studies with nutritional sufficiency. That could diminish the additive effects of GAA on growth performance, as the broilers may not have experienced stress or a nutrient deficit that would typically prompt the body to utilize the GAA more effectively [25]. GAA is a precursor of creatine, which provides advantages for broiler performance and metabolism, especially because the demand for creatine increases substantially when birds are exposed to heat stress. This highlights the beneficial effects of GAA for broilers, particularly under challenging environmental or management conditions. In broilers subjected to heat stress, dietary GAA has been shown to be beneficial due to enhanced muscle creatine loading and its arginine-sparing effects [12,30]. Such conditions may amplify the metabolic advantages of GAA, allowing a clearer distinction of its impact on energy metabolism and overall performance. Further studies should investigate metabolic responses to dietary GAA supplementation to clarify its role and contribution to broiler nutrition under challenging conditions.

## 5. Conclusions

GAA supplementation can be recommended due to the positive effects observed on feed conversion and caloric conversion in the starter phase (1–21 d) of broilers when used. Although nutrient digestibility parameters were not significantly affected by either ME level or GAA inclusion, it was observed that broilers fed PC diets supplemented with GAA showed improved AME and AMEn compared to those fed PC diets without GAA.

## Figures and Tables

**Table 1:** Composition of experimental diets (%).

	Starter Phase (1–7 d)						Grower Phase (8–21 d)						Finisher Phase (22–35 d)					
Metabolizable Energy	PC	PC	−50	−50	−100	−100	PC	PC	−50	−50	−100	−100	PC	PC	−50	−50	−100	−100
Guanidinoacetic Acid	Without	With	Without	With	Without	With	Without	With	Without	With	Without	With	Without	With	Without	With	Without	With
Ingredient, %																		
Corn	49.08	49.08	49.08	49.08	49.08	49.08	56.28	56.28	56.28	56.28	56.28	56.28	56.92	56.92	56.92	56.92	56.92	56.92
Soybean meal (45% CP)	44.61	44.61	44.61	44.61	44.61	44.61	38.09	38.09	38.09	38.09	38.09	38.09	36.95	36.95	36.95	36.95	36.95	36.95
Soy oil	2.41	2.41	1.84	1.84	1.27	1.27	2.36	2.36	1.79	1.79	1.22	1.22	2.98	2.98	2.41	2.41	1.84	1.84
Limestone	0.97	0.97	0.97	0.97	0.97	0.97	0.97	0.97	0.97	0.97	0.97	0.97	0.96	0.96	0.96	0.96	0.96	0.96
Dicalcium phosphate	1.10	1.10	1.10	1.10	1.10	1.10	0.87	0.87	0.87	0.87	0.87	0.87	0.779	0.779	0.779	0.779	0.779	0.779
Common salt	0.510	0.519	0.519	0.519	0.519	0.519	0.496	0.496	0.496	0.496	0.496	0.496	0.471	0.471	0.471	0.471	0.471	0.471
DL-methionine	0.415	0.416	0.416	0.416	0.416	0.416	0.3198	0.320	0.320	0.320	0.320	0.320	0.329	0.329	0.329	0.329	0.329	0.329
Filler	0.100	0.040	0.669	0.609	1.241	1.176	0.1000	0.040	0.669	0.609	1.241	1.176	0.100	0.040	0.669	0.609	1.241	1.176
L-lysin HCL	0.216	0.217	0.217	0.217	0.217	0.217	0.1384	0.138	0.138	0.138	0.138	0.138	0.145	0.146	0.146	0.146	0.146	0.146
L-threonine	0.191	0.191	0.191	0.191	0.191	0.191	0.1093	0.109	0.109	0.109	0.109	0.109	0.102	0.103	0.103	0.103	0.103	0.103
Vitamin premix ^1^	0.150	0.150	0.150	0.150	0.150	0.150	0.120	0.120	0.120	0.120	0.120	0.120	0.100	0.100	0.100	0.100	0.100	0.100
Mineral premix ^2^	0.050	0.050	0.050	0.050	0.050	0.050	0.050	0.050	0.050	0.050	0.050	0.050	0.050	0.050	0.050	0.050	0.050	0.050
L-valine	0.068	0.068	0.068	0.068	0.068	0.068	0.004	0.004	0.004	0.004	0.004	0.004	0.011	0.011	0.011	0.011	0.011	0.011
Choline chloride (60%)	0.075	0.075	0.075	0.075	0.075	0.075	0.050	0.050	0.050	0.050	0.050	0.050	0.050	0.050	0.050	0.050	0.050	0.050
Coban ^3^	0.025	0.025	0.025	0.025	0.025	0.025	0.025	0.025	0.025	0.025	0.025	0.025	0.025	0.025	0.025	0.025	0.025	0.025
Antioxidant (BHT 99%)	0.010	0.010	0.010	0.010	0.010	0.010	0.010	0.010	0.010	0.010	0.010	0.010	0.010	0.010	0.010	0.010	0.010	0.010
Phytase 10,000 FTU/g	0.005	0.005	0.005	0.005	0.005	0.005	0.005	0.005	0.005	0.005	0.005	0.005	0.005	0.005	0.005	0.005	0.005	0.005
Guanidinoacetic acid	0.000	0.060	0.000	0.060	0.000	0.060	0.000	0.060	0.000	0.060	0.000	0.060	0.000	0.060	0.000	0.060	0.000	0.060
Composition calculated																		
ME. kcal/kg	2980	2980	2930	2930	2880	2880	3050	3050	3000	3000	2950	2950	3100	3100	3050	3050	3000	3000
CP, %	23.23	23.23	23.23	23.23	23.23	23.23	20.78	20.78	20.78	20.78	20.78	20.78	20.35	20.35	20.35	20.35	20.35	20.35
Ca analyzable, %	0.77	0.77	0.77	0.77	0.77	0.77	0.70	0.70	0.70	0.70	0.70	0.70	0.67	0.67	0.67	0.67	0.67	0.67
aP, %	0.47	0.47	0.47	0.47	0.47	0.47	0.42	0.42	0.42	0.42	0.42	0.42	0.40	0.40	0.40	0.40	0.40	0.40
Dig Arg, %	1.40	1.40	1.40	1.40	1.40	1.40	1.24	1.24	1.24	1.24	1.24	1.24	1.21	1.21	1.21	1.21	1.21	1.21
Dig Lys, %	1.32	1.32	1.32	1.32	1.32	1.32	1.12	1.12	1.12	1.12	1.12	1.12	1.100	1.100	1.100	1.100	1.100	1.100
Dig Met, %	0.70	0.70	0.70	0.70	0.70	0.70	0.58	0.58	0.58	0.58	0.58	0.58	0.588	0.588	0.588	0.588	0.588	0.588
Dig Met + Cys, %	0.99	0.99	0.99	0.99	0.99	0.99	0.85	0.85	0.85	0.85	0.85	0.85	0.850	0.850	0.850	0.850	0.850	0.850
Dig Thr, %	0.89	0.89	0.89	0.89	0.89	0.89	0.75	0.75	0.75	0.75	0.75	0.75	0.730	0.730	0.730	0.730	0.730	0.730
Dig Trp, %	0.25	0.25	0.25	0.25	0.25	0.25	0.22	0.22	0.22	0.22	0.22	0.22	0.217	0.217	0.217	0.217	0.217	0.217

PC: positive control; ^1^ vit. A: 9.000.000 UI/kg; vit. D: 2.500.000 UI/kg; vit. E: 20.000; vit K3: 2.500; vit. B1: 1.500 mg/kg; vit. B2: 6.000 mg/kg; vit. B6: 3000 mg/kg; vit. B12: 12,000 mcg/kg; niacin: 25,000 mg/kg; biotin: 60 mg/kg; ac. folic: 800 mg/kg; ac. pantothenic: 12,000 mg/kg; Se: 250 mg/kg. ^2^ Cu 20,000; Fe 100,000 mg/kg; Zn 100,000 mg/kg; Mn 160,000 mg/kg; I 2000 mg/kg. ^3^ Monensin was included at 99 mg/kg (Elanco Animal Health, Greenfield, IN, USA).

**Table 2:** *p* values for main effect ME and GAA and interaction ME × GAA in male Ross broilers 1 to 7 d, 1 to 21 d, and 1 to 35 days old fed diets with different levels of metabolizable energy supplemented or not with guanidinoacetic acid.

	*p* Values				
	1 to 7 d				
Effects	BW (g)	FI (g)	BWG (g)	FCR (g:g)	CC (kcal/g)
ME	0.178	0.480	0.115	0.695	0.016
GAA	0.430	0.827	0.390	0.579	0.595
ME × GAA	0.417	0.213	0.345	0.209	0.208
	1 to 21 d				
ME	0.936	0.0154	0.996	<0.0001	0.024
GAA	0.413	0.207	0.592	0.0005	0.0004
ME × GAA	0.289	0.522	0.136	0.029	0.246
	1 to 35 d				
ME	0.417	0.004	0.408	0.083	0.122
GAA	0.473	0.053	0.466	0.207	0.206
ME × GAA	0.454	0.676	0.445	0.945	0.937

ME—metabolizable energy; GAA—guanidinoacetic acid; BW—body weight; FI—feed intake; BWG—body weight gain; FCR—feed conversion ratio; CC—caloric conversion.

**Table 3:** Interaction between metabolizable energy levels and the use of guanidinoacetic acid in the diet of male Ross broilers on FCR at 21 d (means ± standard error).

GAA/ME	PC	−50	−100	*p* Values
With GAA	1.154 ± 0.004 B b	1.184 ± 0.005 a	1.188 ± 0.004 a	<0.0001
Without GAA	1.178 ± 0.003 A b	1.192 ± 0.003 a	1.201 ± 0.009 a	0.005
*p* values	0.0009	0.231	0.068	

ME—metabolizable energy; GAA—guanidinoacetic acid; PC—positive control; FCR—feed conversion ratio. Uppercase letters in columns differ from each other by F-test as a function of GAA use within energy level. Lowercase letters in rows differ from each other by energy level within GAA use.

**Table 4:** Main effect ME in performance of male Ross broilers 1 to 7 d, 1 to 21 d, and 1 to 35 days old fed diets with different levels of metabolizable energy supplemented or not with guanidinoacetic acid (means ± standard error).

Effect ME	CC (kcal/g) 7 d	FI (g) 21 d	FI (g)35 d	CC (kcal/g) 21 d
PC	2.934 ± 0.16 a	1301.3 ± 34.8 b	3669.11 ± 90.09 b	3.546 ± 0.05 ab
−50	2.850 ± 0.12 ab	1327.4 ± 48.0 ab	3702.04 ± 88.35 ab	3.555 ± 0.04 a
−100	2.809 ± 0.14 b	1334.0 ± 33.8 a	3761.28 ± 81.70 a	3.514 ± 0.07 b
*p* values	0.011	0.005	0.002	0.043

ME—metabolizable energy; PC—positive control; FI—feed intake; CC—caloric conversion. Lowercase letters in the columns differ from each other by Tukey’s test at 5%.

**Table 5:** Main effects of GAA inclusion on growth performance of male Ross broilers 1 to 7 d, 1 to 21 d, and 1 to 35 days old fed diets with different levels of metabolizable energy supplemented or not with guanidinoacetic acid (means ± standard error).

GAA	CC (kcal/g) 21 d
With GAA	3.516 ± 0.04 b
Without GAA	3.562 ± 0.05 a
*p* values	0.0003

CC—caloric conversion. Lowercase letters in the columns differ from each other by F-test at 5%.

**Table 6:** *p* values for main effect ME and GAA and interaction ME × GAA for apparent metabolizable energy (AME), apparent metabolizable energy corrected for nitrogen (AMEn), digestibility coefficient (DC) of crude protein, dry matter, Ca and P utilization, and nitrogen balance of Ross male broilers 18 to 21 d old.

Effect	AME (kcal/kg) *	AMEn (kcal/kg) *	DC CP (%)	DC Ca (%)	DC P (%)	DC DM (%)	Balance N (g)
Probabilities							
ME	<0.0001	<0.0001	0.7485	0.016	0.463	<0.0001	0.170
GAA	0.0002	<0.0001	0.9938	0.855	0.429	0.836	0.595
ME × GAA	<0.0001	<0.0001	0.0725	0.010	0.005	<0.0001	0.339

* In dry matter. ME—metabolizable energy; GAA—guanidinoacetic acid; DC—digestibility coefficient; CP—crude protein; Ca—calcium; P—phosphorus; DM—dry matter.

**Table 7:** Interactions between metabolizable energy levels and the use of guanidinoacetic acid in the diet of male Ross broilers on apparent metabolizable energy and apparent metabolizable energy corrected for nitrogen (means ± standard error).

	AME, kcal/kg			AMEn, kcal/kg		
ME/GAA	Without	With	*p* Values	Without	With	*p* Values
PC	3504.2 ± 31.0 a B	3567.2 ± 31.8 a A	<0.0001	3254.8 ± 27.5 a B	3326.4 ± 27.3 a A	<0.0001
−50 kcal	3465.3 ± 13.3 b	3456.5 ± 22.6 b	0.0900	3207.7 ± 15.6 b	3220.2 ± 19.1 b	0.0610
−100 kcal	3370.5 ± 20.1 c	3380.6 ± 17.8 c	0.1944	3128.4 ± 19.0 c	3141.4 ± 19.3 c	0.1487
*p* values	<0.0001	<0.0001		<0.0001	<0.0001	

ME—metabolizable energy; GAA—guanidinoacetic acid; PC—positive control. Lowercase letters in the columns differ by Tukey’s test at 5% effect of ME within each level of GAA (with and without). Uppercase letters in the rows differ by the F-test at 5% effect of GAA (with and without) within each energy.

**Table 8:** Interactions between metabolizable energy levels and the use of guanidinoacetic acid in the diet of male Ross broilers on Ca and P utilization and dry matter digestibility coefficient (means ± standard error).

	Ca Utilization, %			P Utilization, %			DC DM, %		
ME/GAA	Without	With	*p* Values	Without	With	*p* values	Without	With	*p* Values
PC	70.2 ± 2.3 ab	71.8 ± 3.3	0.0818	69.6 ± 4.9 B b	72.8 ± 4.2 A	0.0066	73.2 ± 0.8	73.6 ± 0.6 a	0.0602
−50 kcal	72.9 ± 2.7 A a	69.7 ± 2.6 B	0.0089	75.2 ± 3.3 A a	69.9 ± 5.5 B	0.0044	72.8 ± 0.3 B	73.4 ± 0.7 A a	0.0036
−100 kcal	68.4 ± 4.1 b	69.6 ± 0.8	0.2066	71.3 ± 6.3 ab	70.9 ± 3.8	0.3510	72.9 ± 0.3 A	71.9 ± 0.6 B b	<0.0001
*p* values	0.0004	0.0978		0.0013	0.0663		0.1220	<0.0001	

ME—metabolizable energy; GAA—guanidinoacetic acid; PC—positive control. Lowercase letters in the columns differ by Tukey’s test at 5% effect of MRE within each level of GAA (with and without). Uppercase letters in the rows differ by the F-test at 5% effect of GAA (with and without) within each energy.

**Table 9:** Apparent metabolizable energy (AME), apparent metabolizable energy corrected for nitrogen (AMEn), digestibility coefficient (DC) of crude protein, dry matter, Ca and P utilization, and nitrogen balance of Ross male broilers 18 to 21 days old fed diets with different levels of metabolizable energy supplemented or not with guanidinoacetic acid (means ± standard error).

ME (Kcal/kg)	AME (kcal/kg) *	AMEn (kcal/kg) *	DC CP (%)	DC Ca (%)	DC P (%)	DC DM (%)	Balance N (g)
PC	3535.7 ± 7.8	3290.6 ± 8.0	69.5 ± 0.3	71.0 ± 0.5	71.2 ± 0.8	73.4 ± 0.1	121.5 ± 1.4
−50	3460.9 ± 3.9	3225.5 ± 3.7	69.7 ± 0.2	71.3 ± 0.6	72.5 ± 1.0	73.1 ± 0.13	118.9 ± 0.8
−100	3375.6 ± 3.7	3134.9 ± 3.7	69.8 ± 0.25	69.0 ± 0.6	71.1 ± 0.9	72.4 ± 0.13	121.5 ± 0.8
With GAA	3468.1 ± 12.7	3228.7 ± 13.0	69.7 ± 0.21	70.4 ± 0.45	71.2 ± 0.69	73.0 ± 0.14	121.0 ± 0.90
Without GAA	3446.7 ± 9.8	3205.3 ± 9.1	69.7 ± 0.26	70.5 ± 0.56	72.0 ± 0.82	72.9 ± 0.09	120.3 ± 0.9
Probabilities							
ME	<0.0001	<0.0001	0.7485	0.016	0.463	<0.0001	0.170
GAA	0.0002	<0.0001	0.9938	0.855	0.429	0.836	0.595
Interaction ME X GAA	<0.0001	<0.0001	0.0725	0.010	0.005	<0.0001	0.339

* In dry matter. ME—metabolizable energy; GAA—guanidinoacetic acid; PC—positive control; DC—digestibility coefficient; CP—crude protein; Ca—calcium; P—phosphorus; DM—dry matter.

## Data Availability

Dataset available on request from the authors. The raw data supporting the conclusions of this article will be made available by the authors on request.

## Abbreviations

The following abbreviations are used in this manuscript.

ADC (%)	apparent nutrient digestibility coefficient
AIC	Akaike information criterion
AME	apparent metabolizable energy
AMEn	apparent metabolizable energy corrected for nitrogen balance
ATP	adenosine triphosphate
BW	body weight
BWG	body weight gain
CC	caloric conversion
CP	crude protein
DM	dry matter
FCR	feed conversion ratio
FI	feed intake
GAA	guanidinoacetic acid
ME	metabolizable energy
NC	amount of nutrient consumed
NEP	net energy for production
NEx	amount of nutrient excreted
NexA	amount of nutrient in the excreta
NFA	amount of nutrient in the feed
PC	positive control
RE	reduced energy
SE	standard energy
TFC	total feed consumed
TPEx	total production of excreta
